# “The first transition is from resident to patient”: understanding the decisional needs of long-term care residents preparing for hospital transitions

**DOI:** 10.1093/geront/gnaf272

**Published:** 2025-11-21

**Authors:** Alixe Ménard, Yamini Singh, Lauren Konikoff, Michaela Adams, Daniel Kobewka, Krystal Kehoe MacLeod

**Affiliations:** Department of Medicine, Ottawa Hospital Research Institute, Ontario, Canada; Department of Medicine, Ottawa Hospital Research Institute, Ontario, Canada; Bruyère Health Research Institute, Ontario, Canada; Perley Health Centre of Excellence in Frailty-Informed Care™, Ontario, Canada; Department of Medicine, Ottawa Hospital Research Institute, Ontario, Canada; Bruyère Health Research Institute, Ontario, Canada; Bruyère Health Research Institute, Ontario, Canada; Department of Family Medicine, University of Ottawa, Ontario, Canada

**Keywords:** Long-term care, Hospitals, Decision-making, Patient-centered care, Care transition

## Abstract

**Background and Objectives:**

Long-term care (LTC) residents are frequently transferred to hospitals, yet these transitions do not always align with residents’ care goals or preferences. This study aimed to assess the decisional and informational needs of LTC residents, focusing on their decision-making priorities and knowledge gaps when facing hospital transition decisions.

**Research Design and Methods:**

The researchers conducted 28 semi-structured interviews with residents (*n* = 9), care partners (*n* = 8), and staff (*n* = 11) across three LTC homes in Ontario, Canada. Guided by the Ottawa Decision Support Framework and the Decisional Needs Assessment Workbook, the interviews explored participants’ experiences, decision-making needs, and information requirements for LTC-to-hospital transitions. Interviews were transcribed verbatim and analyzed using reflexive thematic analysis.

**Results:**

Participants were predominantly white and female, with 68.3 as residents’ mean age and 70.5 as care partners’ mean age. Staff (mean age: 46.5) were more ethnically diverse in a range of clinical and leadership roles. Four interconnected themes about resident needs during LTC-to-hospital transitions emerged: (a) communication and transparency, (b) continuity of care between LTC homes and hospitals, (c) awareness of ageist assumptions and conflicting priorities, and (d) trust building during care transitions.

**Discussion and Implications:**

This study highlights the persistent challenges that residents, care partners, and staff face in preparing for and making decisions regarding LTC-to-hospital transitions. The findings emphasize the urgent need for decision-support tools that empower LTC residents to feel better prepared to make decisions that reflect their values, priorities, and goals of care.

##  

Care transitions refer to the movement of patients from one healthcare setting to another (Naylor & Keating, 2008). In the long-term care (LTC) sector, transitions between LTC homes and the hospital can occur in response to infections, falls, fractures, and cardiovascular events that cannot be adequately managed in LTC (Gruneir et al., 2010). Transitions from LTC to hospital can also occur due to general functional decline, changes in mental status, or uncertainty among family and staff regarding whether a resident requires hospital-level care (Yin et al., 2025). While hospital transitions can offer benefits, such as rapid access to diagnostic testing and treatments that may address symptoms and potentially prolong life (Gruneir et al., 2010), the care provided in emergency rooms is often not tailored to the needs of older adults and can, in some cases, result in poor health outcomes, including increased morbidity and mortality (Payne et al., 2023). LTC-to-hospital transitions can also cause harm including hospital-acquired infections, delirium, and distress associated with leaving one’s familiar LTC environment for an unknown place (Mitchell et al., 2018). Further, LTC-to-hospital transitions may not align with residents’ preferences or goals of care, especially in cases where residents may prioritize comfort over life-prolonging measures (Nemiroff et al., 2019). Good transitions in care are also dependent on resources, structure, integrated care approaches across settings, multidisciplinary collaboration, and information continuity (Fakha et al., 2023).

The ethical principle of autonomy (i.e., honoring an individual’s right to make their own choices) supports residents’ involvement in decisions about their health, including whether or not to be transferred to hospital (Bosisio & Barazzetti, 2020). This foundational principle in healthcare is considered by patients to be a sign of respect, dignity, and human value (Moilanen et al., 2021). Yet, in LTC, where residents often depend on others for daily care, a paternalistic culture has emerged that narrowly equates autonomy with individual capacity, leading to the mistaken assumption that residents with lower competence have less say in their care (Moilanen et al., 2021).

Involving residents and care partners in transition decisions enhances quality of care and results in better decision-making (Cranley et al., 2020). Moreover, meaningful engagement is central to person-centered care, which promotes dignity and respect and challenges the traditional medicalization of older adults in LTC, where they often lack control over their own care (Eaton, 2016; Thompson et al., 2018). In the context of care transitions, a person-centered approach involves providing LTC residents and care partners the opportunity to engage in decision-making processes alongside their care providers (Eaton, 2016).

Care planning processes, including goals of care conferences, play a crucial role in establishing the preferences of LTC residents and ensuring that their care aligns with their values and needs (Tinetti et al., 2021). Goals of care conferences, typically scheduled shortly after residents move into LTC, are discussions that aim to clarify residents’ wishes regarding various care decisions, such as do-not-resuscitate orders, hospitalization preferences, and symptom management (Cranley et al., 2020). These types of processes support LTC residents in understanding and considering the risks and benefits of major decisions about their care and the potential outcomes of these decisions (Eaton, 2016). However, even with these processes in place as part of the admission of residents to LTC, research shows that residents still struggle to feel engaged in decision making about their care (Lynch et al., 2022). This is especially problematic during acute crisis points, such as a transition decision, because of the emotional and time-sensitive nature of these decisions and the potential for decline in health and functioning (Freeman et al., 2017; Reid et al., 2013). Decisional regret is also a concern when there is a mismatch in preferences or conflict between the resident and substitute decision-maker, especially if the outcome leads to increased caregiver burden or reduced resident well-being (Lognon et al., 2022).

The Ottawa Decision Support Framework defines a quality decision about a potential hospital transition as one in which the resident is well-informed, actively engaged, and supported in aligning decisions with their values (Stacey et al., 2020). This involves providing residents and care partners with information about both the risks and benefits of transitions to ensure their decision is evidence-based and reflects their personal preferences. This framework conceptualizes decisional need as a deficit that can reduce the quality of a decision (Stacey et al., 2020). Decisional needs may relate to (a) decisional type (e.g., having multiple options, uncertain outcomes, or outcomes that weigh differently depending on the decision-maker’s values) and (b) decisional timing (e.g., decisions that are urgent, delayed, or unpredictable). According to Stacey et al. (2020), decisional needs are influenced by available knowledge, expectations, values, supports and resources, and personal and clinical considerations. Decision-makers may become unreceptive when they are no longer open to receiving information or deliberating between options. They experience decisional conflict when they express uncertainty due to perceived risk, potential regret, or challenges to their personal values (Stacey et al., 2020).

Despite efforts to improve care coordination and continuity across health care settings, the transition from LTC to hospital remains particularly complex and challenging due to poor communication between healthcare staff and residents, and incomplete information transfer across care settings (Naylor et al., 2009). These challenges can leave residents and care partners feeling overwhelmed and unsupported in making time-sensitive decisions (Mitchell et al., 2018).

Guided by the Ottawa Decision Support Framework (Stacey et al., 2020), which conceptualizes decisional need as deficits in knowledge, values clarification, or support that can compromise decision quality, the researchers aimed to assess the informational and decisional needs of LTC residents, care partners, and staff regarding LTC-to-hospital transition decisions. Although processes such as goals of care conferences exist to support resident decision-making, evidence shows that residents and care partners often remain insufficiently engaged, particularly during acute or time-sensitive transitions (Cranley et al., 2020). Moreover, little is known about the specific knowledge gaps and support needs that influence preparation for and experiences of these transitions. This project is part of a 4-year study to co-design a decision-making tool to support resident-centered decisions about LTC-to-hospital transitions at the onset of illness or injury (Ménard et al., 2024).

## Methods

### Study design

In this exploratory-descriptive study, the researchers conducted a qualitative needs assessment using semi-structured interviews with LTC residents, care partners, and staff. This study followed a constructivist approach, which recognized participants’ experiences as subjective and shaped by their individual perspectives (Whitman, 1993). This approach was appropriate for understanding residents’ decision-making needs as it enabled the research team to explore how the personal experiences of residents, care partners, and staff members over time with issues such as care planning, interactions with others, and their own health care experiences shaped their current views of LTC-to-hospital transitions. Even though these views were highly personal, this approach allowed the research team to co-construct cross-cutting themes to develop a shared understanding of residents’ needs in this specific context.

### Study sample and inclusion criteria

The researchers used purposive sampling to recruit participants from three partnering LTC homes in Ottawa, Ontario, Canada. Resident, care partner, and staff participants were recruited through presentations at their respective council meetings, where the research team introduced the study and invited attendees to sign up for interviews. Recruitment posters were also displayed in hallways, and study advertisements were included in newsletters. In addition, two research team members (AM & YS) set up a table and presentation poster at two of the LTC homes to engage potential participants directly in the hallways through informal discussions. Two research team members (AM & MA) also attended a Cultural Awareness and Inclusion Committee meeting at one of the participating homes to gather insights on recruiting diverse perspectives. Recruiting care partners proved challenging, prompting AM to reach out to the provincial Family Council, which expanded the recruitment to include perspectives beyond the three local LTC homes. The LTC homes engaged are referred to henceforth as Site 1, Site 2, and Site 3, and the provincial Family Council as Site 4.

Interviewees were required to be fluent in either French or English to participate. Resident participants were eligible if they had experienced a transition from LTC to hospital within the past year and were able to discuss their care and provide consent or ongoing assent. Care partner participants were eligible if they had been involved in the decision-making process regarding a resident’s LTC-to-hospital transition. Staff participants were eligible if they had direct involvement in LTC-to-hospital transitions. All eligible participants were invited to participate in a 1-hour interview, which could be conducted in-person, virtually (via Microsoft Teams), or by telephone, depending on their preference. Interviews were conducted between November 2023 and April 2024. All participants provided oral or written consent before the interview and received a $25 gift card as a token of appreciation for their participation. This study was approved by the Bruyère Health Research Ethics Board (#M16-23-036).

### Positionality

The researchers recognize that their backgrounds and experiences inevitably shape how they view and approach this study. Their team includes research coordinators and associates (AM, YS, LK, and MA) whose work primarily focuses on LTC, a physician with direct experience providing care to LTC residents (DK), and a postdoctoral fellow with expertise in equity-informed healthcare for older adults (KKW). Due to this sustained focus, the researchers bring a particular perspective on LTC that offers deep contextual knowledge but also risks narrowing their lens. They also acknowledge the power dynamics at play when interviewing LTC residents, who are often marginalized in decision-making about their own care. To address these dynamics and remain reflexive in their interpretations, they held weekly team meetings to discuss potential biases and relational awareness. These discussions helped them monitor how their positionalities may have influenced data collection and analysis, while working to ensure that findings remained grounded in participants’ own voices.

### Data collection

The researchers’ interview guide was theoretically grounded in the Ottawa Decision Support Framework and informed by the Decisional Needs Assessment Workbook by Jacobsen et al. (2013). These tools provided the basis for identifying and categorizing the factors that influence decision-making, such as informational needs, values clarification, and decision support. Notably, the Ottawa Decision Support Framework guides researchers in assessing participants’ decision-making needs, developing decision support interventions, and evaluating their effects on decision outcomes (Stacey et al., 2020). It supports the assessment of decisional needs by exploring factors such as timing, conflict, knowledge gaps, and support requirements from the perspectives of residents and care partners (Stacey et al., 2020). The framework also evaluates decision outcomes across three domains: (a) decision quality, ensuring choices are informed and aligned with personal values; (b) the decision-making process, through addressing identified needs; and (c) impact, by measuring the resulting intervention’s uptake and application in real-world clinical settings (Stacey et al., 2020). Similarly, the Decisional Needs Assessment Workbook, developed by the same team of experts, outlines key questions to identify opportunities for decision support, such as: *What is currently being done? How are these decisions typically made? How do practitioners support clients’ decision making? What are the barriers and facilitators to providing this support?* These prompts informed the development of the interview questions, which explored the decision-making processes of residents, care partners, and staff during LTC-to-hospital transitions, as well as the information they perceived necessary to make informed choices (Jacobsen et al., 2013).

As a result, the researchers’ interview guide (see online supplementary material), explored participants’ experiences with hospital transitions during their time in LTC, including past hospital visits, the presence and use of advance care plans, involvement in decision-making processes, perceived benefits and challenges of transitions, and recommendations for enhancing decision support. Interviews were conducted by one of two team members (AM or MA), audio-recorded, de-identified, and transcribed verbatim by a professional transcription service. Demographic information was collected via a questionnaire to contextualize participants’ responses and capture relevant background characteristics.

### Data analysis

Interview transcripts were analyzed using reflexive thematic analysis (RTA), a flexible and iterative approach that emphasizes researcher subjectivity and the co-construction of meaning through engagement with the data (Braun & Clarke, 2019). RTA was favored because it explicitly acknowledges researcher positionality in the analysis of interviews and recognizes that coding is an interpretative, iterative process rather than a purely objective or mechanical task (Braun & Clarke, 2023). The researchers followed Braun and Clarke’s six-phase process: (a) familiarization with the data, (b) generating initial codes closely aligned with the research question, (c) constructing initial themes, (d) reviewing and refining themes, (e) defining and naming themes, and (f) producing the final report. All interviews were double coded (by AM and YS or AM and LK) early in the process to support consistency in coding approaches and deepen team discussions, recognizing that in RTA, reliability is conceptualized through reflexivity rather than coder agreement (O’Connor & Joffe, 2020).

Following each interview, the interviewer (AM or MA) completed a structured self-reflexive form to document contextual details, key insights, emerging themes, and reflexive observations. The form included sections to describe the setting and participant background (e.g., professional role, rapport), summarize poignant insights relevant to the research question, identify patterns emerging across interviews, and flag considerations for data collection. Interviewers also reflected on their assumptions, positionality, and potential biases, as well as practical or ethical dilemmas raised during the interview. These forms were used to enrich data interpretation, support team reflexivity, and ensure transparency in the co-construction of meaning. These entries were analyzed alongside interview transcripts to enrich interpretation and contextualize how researcher perspectives may have shaped data generation and analysis.

For example, early codes such as “going to the hospital alone,” “residents feeling powerless,” and “staff overwhelmed by paperwork” were gradually clustered into broader themes like “gaps in communication” and “systemic constraints on decision-making.” Similarly, codes capturing “empathy from staff,” “positive staff encounters,” and “building rapport with families” informed the development of a theme on “trust building during care transitions.” This iterative theme-building process required multiple rounds of refinement, where the researchers compared coded extracts across participants to check for consistency and depth.

To ensure trustworthiness and rigor in the study, the researchers drew on Lincoln and Guba’s (1986) four criteria: credibility, dependability, confirmability, and transferability. Credibility was supported through peer debriefing and weekly team meetings (AM, MA, LK, YS, and KKM) during which coding decisions, emerging themes, and interpretive insights were discussed (Lincoln & Guba, 1986). They further engaged the broader research team, including care provider partners, to assess whether findings resonated with field-based experiences. Dependability was fostered by maintaining a detailed audit trail of methodological decisions across data collection and analysis. They also presented the results to residents from Site 1 and had detailed discussions about accuracy in the reporting of their experiences. Confirmability was enhanced through reflexive journaling and integrating interviewer reflections into the analytic process (Doyle, 2013). Transferability was supported by collecting rich, descriptive data across three LTC homes, enabling exploration of shared and divergent perspectives on LTC-to-hospital transitions across multiple contexts (Lincoln & Guba, 1986).

## Results

### Participant demographics

A total of 28 interviews were conducted across three LTC homes in Ottawa, in both English and French. Participants included 9 residents, 8 care partners, and 11 staff members (e.g., personal support workers, care coordinators, nurses, and physicians). Among residents, most were women (77.8%) with a mean age of 68.3 years, and all identified as white. The care partner group was also predominantly women (62.5%), with a mean age of 70.5 years and primarily white backgrounds (87.5%). Staff participants were more ethnically diverse and mostly women (90.9%), with a mean age of 46.5 years. They represented a range of roles from direct care to leadership positions. Table 1 provides a detailed summary of participant characteristics.

**Table 1.** Participant demographics.

Characteristics	Residents (*n* = 9)	Care partners (*n* = 8)	Staff (*n* = 11)
**Age**	68.33	70.50	46.45
**Male**	2 (22.2%)	3 (37.5%)	1 (9.1%)
**Female**	7 (77.8%)	5 (62.5%)	10 (90.1%)
**Ethnicity**			
** Racialized minority**	−	1 (12.5%)	8 (72.7%)
** White**	9 (100%)	7 (87.5%)	3 (27.3%)
**Education**			
**High school or less**	2 (22.2%)	1 (12.5%)	−
**Some post-secondary**	3 (33.3%)	3 (37.5%)	3 (27.3%)
**University degree or higher**	4 (44.4%)	4 (50.0%)	8 (72.7%)
**Yearly household income**			
**Low income (<$35,000)**	5 (55.6%)	−	−
**Middle income ($35,000–$74,999)**	2 (22.2%)	1 (12.5%)	3 (27.3%)
**High income (≥ $75,000)**	−	6 (75.0%)	8 (72.7%)
**N/A (data not provided)**	2 (22.2%)	1 (12.5%)	−

*Note*. N/A = not applicable.

### Themes

The thematic analysis revealed four themes that describe the decision support needs of LTC residents as they pertain to hospital transitions: (a) a need for communication and transparency; (b) a need for continuity of care between LTC homes and hospitals; (c) a need to address ageist assumptions and conflicting priorities, and (d) a need to build trust during care transitions.

#### Theme 1: a need for communication and transparency

Concerns about the appropriateness and frequency of hospital transitions were raised by several participants, underscoring a perceived lack of clarity around clinical decision-making processes in LTC, particularly as communicated to residents when their health needs changed. Residents and care partners described feeling excluded from these decisions, which often led to distress and a sense of powerlessness. One resident critically reflected on the rationale behind such transitions, questioning their necessity and the need for escalation to hospital:

My first issue is really to question the transitions to hospital, how many there are, how frequently it happens, for what purpose it happens. And I think there’s a lot of it happening unnecessarily. I mean, obviously, if someone’s unconscious, staff are going to call an ambulance. They’re going to take them away. I get that. If, however, I become ill and have an infection, I don’t have to go to the hospital to get testing done, I don’t think. If I have to have bloodwork, why does that require a hospital move? Why does that require sending me to the hospital for bloodwork? They [LTC staff] really send you to the hospital as soon as they’re uncomfortable with attending to what’s going on with you. And I don’t know what that threshold is. (Resident A from Site 1, age 68)

While some residents questioned the rationale behind hospital transitions, others described a sense of deference to staff decisions due to a lack of information about their own condition or care options. During the interviews, participants were asked whether they felt they had a role in the decision about whether to go to the hospital. Many residents indicated they did not, often citing staff’s superior access to clinical information as the reason:

If they think I have to go to the hospital, I’m not going to say no. They know more about what the circumstances are than I myself, and if they say go, well, even though I don’t want to go, they know if it’s best for me to go, you see? If they decide for me to go to the hospital, I will not hesitate. My answer will always be yes. (Resident B from Site 2, age 66)

Participants also highlighted the complexity and unpredictability of hospital transition decisions, noting that the need for hospitalization often depended on specific medical situations, which were not always anticipated or discussed in advance. One participant reflected on the challenges of planning for various scenarios:

How could you possibly list every permutation that would come up? Like, if you need bloodwork, do you want to go to the hospital? I probably would have said no. If you need a blood transfusion, do you want to go to the hospital? Yeah, right? If your kidneys fail, do you want to go to the hospital? Maybe. Yeah. If you have a heart attack, do you want to go to the hospital? Yeah. But like, describe the situations where you would be agreeable to being sent to the hospital. No, I don’t remember a conversation like that. (Resident A from Site 1, age 68)

In addition, participants described a lack of clarity around hospital transition plans, with both residents and care partners unaware of the required documentation and costs related to these transitions.

My wife got a call here that [my mom] was going [back to LTC]. We were going to need to pay the $200 ambulance fee for the return trip. But at first, I wasn’t aware that the ambulance, at first [LTC to hospital], is covered by Ontario or the City of Ottawa. So we’ll see how things evolve. If the bill comes by mail or whatever, we’ll find out. So there you have it, we didn’t know. My mom was also in isolation for several days when she came back from the hospital. I don’t know if it’s because in the section she was in, there had been some kind of COVID or flu, but she was in isolation. She stayed in isolation when she came back. In fact, for four days. But it wasn’t clear why. And even then, she told me, it was protocol but we didn’t know. (Care partner A from Site 3, age 64)

This theme demonstrates that hospital transitions in LTC can occur without clear communication or transparency, leaving residents and care partners uncertain about the rationale behind decisions, the implications of transitions, and their role in the process. This not only reinforces a sense of exclusion from care planning and a lack of informed participation, but also perpetuates existing power dynamics in which staff are positioned as knowing what is best, thereby undermining the principles of resident-engaged care. While residents and care partners consistently described exclusion from hospital transition decisions, staff tended to emphasize their clinical responsibility and need to act quickly in urgent situations. This created a tension: What staff viewed as timely decision-making was often experienced by residents and care partners as a lack of transparency and communication.

#### Theme 2: a need for continuity of care between LTC and hospitals

Participants identified significant gaps in continuity of care during hospital transitions, particularly due to breakdowns in communication of crucial medical information between settings. A major concern was the lack of clear and timely documentation of medications, which created challenges in managing residents’ treatment, especially pain, upon arrival at the hospital. As one care partner explained:

If you order an ambulance, doesn’t it make sense that you go and get them the list of meds or photocopy it to go with the resident? Because at the hospital, they don’t know what to give the resident if they’re in a lot of pain because they haven’t got what they’re already on, they don’t have the last times that they had this medication, and now that the situation has changed, should they have it again, as a PRN. It leaves a big gap if you don’t have the medications and the times that they were delivered. So that was very frustrating. (Care partner B from Site 1, age 77)

Many participants noted a lack of communication between LTC and hospital settings, where both institutions failed to adequately understand and address the unique needs of LTC residents. One participant emphasized that challenges existed at every stage of the transition process, from the initial hospital transition to the return to LTC, underscoring the need for better coordination between care settings. Reflecting on these challenges, one resident described the profound physical and emotional disruptions experienced during their LTC-to-hospital transitions:

The first transition is from resident to patient. When you leave here, you’re a resident. When you get to the hospital, you’re a patient. And a lot of people don’t know what that means. For me, the reason why I refuse to go to hospital until it’s absolutely necessary, is that they can’t care for me in the hospital. I have no way of getting out of bed. I go in an ambulance, so I don’t have my wheelchair. They have no way of getting me in and out of bed. And their beds do not raise and lower. The level of care is different because in long-term care, you’re being attended to by a staff of PSWs [personal support workers]. And when you go to the hospital, you’re not familiar with any of them and they are unfamiliar with you. And continuity of care is probably one of the most important things to people who live in long-term care because it’s where they find their comfort. (Resident A from Site 1, age 68)

Participants expressed a deep sense of discomfort and fear regarding hospital environments, often shaped by previous experiences or widespread concerns about the quality of care in hospitals. This discomfort led some residents to actively resist transitions to hospitals, preferring to remain in the familiar and more comfortable setting of their LTC home, even when faced with serious health concerns. One resident shared a poignant reflection on their decision to refuse hospitalization, emphasizing their desire to maintain control over their care and avoid the perceived indignities of hospital treatment:

I’ve had a few problems with my heart, and the ambulance came, and they wanted to take me. But I said, I’ve heard so much about what goes on in the hospital. They just put you in the hallway and patients had to come back here. They hadn’t been changed or fed. And so I said, if I’m going to die, I’d rather die here at my home than somewhere strange. And eventually they talked to me about it again. If I’m sick, and if I want to go to the hospital, and I said no. I refuse to go. I’m happier here, and I don’t want to die in a hallway. (Resident B from Site 2, age 78)

This theme demonstrates that poor continuity of care between LTC homes and hospitals is perceived to stem from breakdowns in communication across care settings. Participants’ accounts reflect a desire for greater coordination and mutual understanding between providers, as well as a need to preserve continuity in the relationships and routines that shape everyday life in LTC. Care partners largely emphasized the logistical breakdowns (e.g., missing medication lists, incomplete documentation), while residents emphasized the personal and emotional disruption caused by moving between care settings. Staff, meanwhile, described the challenge of balancing their responsibility to stabilize residents with the practical limitations of coordinating with hospitals. These differing stresses suggest that what staff saw as systemic barriers were experienced by residents and care partners as failures of continuity and compassion.

#### Theme 3: a need to address ageist assumptions and conflicting priorities

Participants described tensions that emerged when residents’ or care partners’ preferences for care did not align with staff priorities, particularly when residents declined hospitalization connected to their preference for comfort versus curative care. These tensions were often shaped by underlying ageist assumptions about whose priorities mattered most and what constituted appropriate care. One staff member, a personal support worker, emphasized a belief in the prioritization of life-prolonging treatment, even when residents expressed a desire to avoid hospital transitions:

Sometimes the family, they’re aware, they’re involved in the decision. They say no, I don’t want to send the resident to hospital. But sometimes, we make it clear that we want them to go to hospital because life has to be prolonged. I’ve heard residents say no, no, no, I’m not going to waste my time at the hospital- both sides, the families and the residents will say that. But we don’t see that. It’s just about saving life. (Care staff A from Site 3, age 61)

While LTC staff often prioritized life-prolonging interventions, some care partners emphasized quality of life, leading to tensions between these two groups during hospital transition decisions. Power of attorney decision-makers were sometimes hesitant to authorize aggressive treatment for the resident in their care, contributing to disagreements with staff. In addition, some residents expressed a strong desire to make their own healthcare decisions but felt excluded from the process. Several participants noted that care planning and hospital transition decisions were often conflated with do-not-resuscitate orders, leading to confusion about the full range of treatment options.

When we talked to the nurse and the doctor about it at the last meeting in September, I think that’s when we decided we weren’t going to resuscitate her. Maybe it was implied that if it was…in the sense that…if she’s already old and inanimate, that we weren’t going to take her to hospital. (Care partner C from Site 3, age 64)

This misalignment between priorities was further compounded by ageist assumptions in care planning, particularly regarding the perceived inevitability of decline in older residents. One resident reflected on their experience with a care conference, expressing concern that the focus was disproportionately placed on their end-of-life wishes rather than their current health needs, despite having recovered from the issue that initially led to hospitalization:

There was no preamble. Like, this was supposed to be my care conference, and I had looked at my chart, and there were some changes that I wanted to be made, but the focus, at least from the point of view of the doctor, was on my last wishes. And I’d been thinking, well, I just got here. As far as I know, the health problem that took me to the hospital initially is cleared up. I haven’t heard anything else that I’m on my way out. But the focus, extensive focus, was on my last wishes, and I found it disconcerting. I wonder how other people would have heard it. You know, I’m cognizant and aware, but what would happen if you were not aware or if you were an extreme worrier? (Resident C from Site 1, age 81)

Moreover, discussions about hospitalization often centered solely on resuscitation decisions rather than broader care planning, leaving gaps in communication about residents’ overall goals of care.

Oh, well, they did discuss transitions to hospital at the care conference that was held shortly after I arrived. And it seemed to me, the purpose of that particular aspect of the interview was to get my feelings around resuscitation and, you know, did I want to be moved to the hospital if I was dying? Did I want to stay at the [LTC home] or be moved to the hospital? (Resident C from Site 1, age 81)

These concerns were echoed by one participant, who noted that LTC staff sometimes relied on ageist assumptions when making care decisions, specifically, assuming that an older resident’s symptoms were simply a natural part of aging rather than signs of a treatable illness. This led to a lack of appropriate medical follow-up and dismissal of the resident’s changing condition. As the participant explained:

I’m a nurse, I know a little bit about what it’s like, whether it’s beneficial or unhealthy to go to the hospital, but the mere fact is that, if it’s an illness that’s been triggered in the last few weeks, it’s not an old illness that’s lying around. Then you can’t just say oh she’s old, she’s going to die. There’s a lot of prejudice in long-term care…ageist prejudice in there because even all the young people and the PSWs [personal support workers], they’re inclined to have prejudices. Rare are those who don’t have any. (Care partner D from Site 4, age 76)

This participant emphasized that the presumption of “she’s old, she’s going to die” reflects an ageist mindset that can lead to fatalism and the under-treatment of new, potentially reversible conditions. This theme reflects resident concerns about being excluded from hospital transition decisions and care planning. Both residents and care partners noted that discussions often focused narrowly on end-of-life decisions, rather than broader goals of care, and raised concerns about ageist assumptions (e.g., viewing older adults’ symptoms as an inevitable part of aging rather than signs of treatable conditions, prioritizing life-prolonging treatment over comfort care, and prematurely focusing on end-of-life planning) shaping how care needs were assessed and addressed. This theme illustrates a clear misalignment whereby staff often framed their role as preserving life through hospitalization, while some care partners prioritized comfort and dignity, and residents sought autonomy in decision-making.

#### Theme 4: a need to build trust during care transitions

Participants identified systemic issues and a lack of trust in both LTC and hospital systems, which influenced decision-making around hospital transitions. Some care partners and residents expressed distrust toward LTC staff due to previous experiences of suboptimal care, shaping their perceptions of when hospitalization was warranted. Conversely, hospital staff were sometimes perceived as dismissive of LTC residents, assuming their needs should be managed within the LTC home rather than requiring emergency intervention. As one resident recounted:

I packed a little bag, a few things, and I took my tablet and everything, and I went there. Sat there for hours in the waiting room, and I finally got into the observation room, and I was waiting. And the ER doctor, as she was ranting—okay, she was young, and she was like, “What do you mean she’s got a pressure sore? She’s in long-term care. They should be able to take care of it. Why can’t they? And why is she in my ER?” The curtain is right there. I can hear. They were saying like, well, she lives in long-term care. Why can’t they take care of that? (Resident E from Site 3, age 55)

Frequent staff turnover in LTC led to inconsistent care and unmet resident needs, heightening feelings of instability in care routines and deepening distrust toward unfamiliar staff. As one care partner explained:

Sometimes there’s a lot of [staff] turnover [in LTC]. There were times when it was stable. And now I see that there’s more rotation. It takes time, it takes re-explaining. Now, I’ve put up a whiteboard in [mother’s] room to indicate her preferences, a bit like guidelines when people come to replace staff at the end of the week. (Care partner F from Site 3, age 64)

In addition to staff turnover, participants noted that limited LTC staffing ratios, particularly overnight, negatively impacted the delivery of person-centered care, leaving residents feeling unsupported and uncertain about the expertise available to manage their health concerns. This contributed to a heightened fear of undesired hospitalization, as illustrated by one participant:

If I’m having some trouble breathing…how comfortable are the staff to deal with it? If the RPN [registered practical nurse] is uncomfortable—and I get that it could quickly exceed their level of expertise. But there is supposed to be an RN [registered nurse] on at nights. Overnight, there’s one RN for an entire building, so that becomes problematic. I don’t know how comfortable the RNs are because, you have to understand, they, by and large, spend a great percentage of their day looking at wounds, attending to assessments, and those kinds of things. They are very seldom reacting to critical incidents. So I don’t know what the threshold is. I just know for myself, they’re very quick to suggest I go to the hospital. (Resident A from Site 1, age 68)

This theme highlights how residents and care partners navigated care transitions amid uncertainty and systemic gaps. Residents and care partners frequently described mistrust in both LTC and hospital staff, citing dismissiveness and turnover. Staff, however, emphasized structural barriers such as limited staffing ratios and high turnover as drivers of these experiences. This reveals a gap: What staff framed as systemic constraints were experienced by residents and families as personal failings of care, undermining trust in both systems.

## Discussion

This study explored the experiences of LTC residents, care partners, and staff during transitions from LTC to hospital, revealing key challenges related to communication, continuity of care, decision-making, resource limitations, and systemic distrust. Four themes emerged, each reflecting a distinct area of need. First, participants emphasized the importance of clear, transparent communication, noting confusion around hospital transition practices, financial implications (e.g., ambulance fees), and return protocols such as needing to isolate. Consistent with previous research, this finding shows that poor communication during transitions between care settings leads to patients and care partners feeling neglected and poorly cared for by medical providers (Mitchell et al., 2018). Prior research has also emphasized the importance of accessible and comprehensive information in supporting decision-making and reducing avoidable transitions (Marincowitz et al., 2022). Notably, communication to residents and care partners that hospital transitions do not guarantee improved outcomes is essential (Marincowitz et al., 2022). By ensuring residents and families are well informed about the risks and benefits of transitions earlier in the care trajectory, it helps to support transition decisions during acute events to be more intentional and person-centered (Trahan et al., 2016).

Second, the participants called for improved continuity of care between LTC homes and hospitals, citing issues such as medication delays and unclear discharge processes (e.g., isolation) that disrupted residents’ well-being. Prior research suggests that continuity of care improves health outcomes and overall patient care satisfaction (King et al., 2022), and without it, patients face increased risk of polypharmacy and medical errors (Lei et al., 2021). Yet, most research on continuity of care does not consider its importance in LTC settings where residents are especially in need of informational (i.e., information to make decisions), relational (i.e., relationships between client and provider) and management (i.e., overall management of care) continuity (King et al., 2022). Continuity of care can be disrupted by a disconnect between LTC and hospitals, partly due to misconceptions about who is responsible for care during transitions (Marshall et al., 2016). While LTC residents technically have access to 24/7 on-call physician coverage for urgent or emergent issues, these physicians are often off-site and responsible for large patient rosters limiting their ability to provide timely, individualized care during transitions (Marshall et al., 2016). Overnight, this challenge is compounded by lower staffing ratios and gaps in personal support worker (PSW) training, which can delay the recognition and escalation of resident health concerns (Hodge et al., 2023). Some studies suggest there may also be hesitancy among night staff to contact physicians after hours (Hastings et al., 2007; Li et al., 2020), which could contribute to the higher number of hospital transfers observed during these periods, a point that warrants further exploration.

While LTC homes typically coordinate residents’ primary care, hospitals are jurisdictionally responsible for providing secondary care in the event of an acute health event (Jones et al., 2022; Kobewka et al., 2020). It is paramount that both care settings work together as interdisciplinary teams with the common goal of providing care to a patient in need of medical attention, yet communication gaps continue to impact continuity of care at a systems level with adverse impacts for resident care during transitions (Carbone et al., 2023; Naylor et al., 2009).

Third, participants identified the need to address ageist assumptions and conflicting care priorities, particularly when preferences for comfort-focused care were overshadowed by a care provider’s bias toward life-prolonging interventions. LTC homes serve individuals with diverse health conditions and care needs, of which some may benefit from palliative care interventions, while others may be suited for more aggressive treatment approaches (Liu et al., 2024). In a resident-centered model of care, the decision between these options lies with the resident and their substitute decision-makers. Yet, power dynamics among care providers, residents and care partners continue to shape discussions about care decisions, particularly in how a resident’s options are presented by a trusted care provider such as a physician, for example, if they favor aggressive measures over comfort-oriented care (Halpern et al., 2013). Unequal power relations between residents, care partners, and LTC staff can exclude residents from participating in their own care decisions when others pursue the outcomes they believe to be in the resident’s best interest (Marincowitz et al., 2022). Addressing these dynamics requires intentional efforts to challenge ageist assumptions, ensure balanced presentation of care options, and uphold residents’ autonomy as central to resident-centered decision-making during transitions (Doherty et al., 2020).

Finally, residents and care partners described a lack of trust in both LTC and hospital staff, often shaped by past negative experiences. Research suggests that trust is dependent on communication and collaboration in LTC, especially given the context wherein many residents rely on their LTC staff and care partners for their basic needs to be met (Hunter et al., 2023). Hunter et al. (2023) suggest that LTC residents and care partners may accept suboptimal care or exclusion from decision-making because of the fear of retaliation if they speak up, and because they recognize that care staff, both in LTC and in hospitals, are overworked. Yet, trust, particularly the kind that allows residents and care partners to voice concerns, can be cultivated through meaningful consideration of care preferences and meaningful collaboration in decision-making (Hunter et al., 2023). Trust is further built on transparency in order to replace fear with a sense of inclusion (Holahan et al., 2022).

### Strengths and limitations

A key strength of this study was the inclusion of triadic perspectives from residents, care partners, and staff across three LTC homes. This comprehensive approach enabled us to capture a nuanced and multifaceted understanding of the LTC-to-hospital transition process, providing a rich description of the complexities involved. In addition, by conducting interviews in both English and French, the researchers incorporated linguistic diversity within the sample, broadening the inclusivity of the participant base (Bouchard & Desmeules, 2013). This bilingual approach also strengthens the transferability of the findings, particularly within the Canadian context (Dash et al., 2022). However, this study’s limitations also warrant discussion. The demographic homogeneity of the resident and care partner participants, most of whom were white, reflects the populations of the LTC homes with whom they were partnered but does not capture the diversity of Canada’s broader population. This limits the applicability of the findings to LTC settings serving ethnically and culturally diverse residents. For example, Indigenous, Black, Asian, and other racialized older adults often encounter unique barriers during care transitions linked to their positionality as members of ethno-cultural minority groups (Chamberlain et al., 2024). These barriers can include language discordance, lack of culturally appropriate care or social and recreation activities, or culturally specific expectations for care and family involvement. This is a missing perspective in the data, given the composition of the sample, resulting in a probable oversimplification of the complexity of transitions from LTC to hospital for residents from non-Western backgrounds. Gender diversity is also underrepresented in the sample; the experiences of men, women, and gender-diverse residents differ in terms of health trajectories, caregiving dynamics, and access to supportive services (Zygouri et al., 2021). Without these perspectives, this analysis does not fully account for how intersecting identities shape decision-making, communication, and experiences of hospital transfers in LTC. There is a need for more work rooted in an intersectional approach and conducted in LTC homes with ethno-culturally diverse resident populations to fill this research gap. In addition, there is the potential for self-selection bias, as individuals with particularly strong views or experiences regarding LTC-to-hospital transitions may have been more likely to participate (Alarie & Lupien, 2021). The researchers’ use of self-reflexive forms after each interview supported their ongoing commitment to dismantling hierarchies of power, particularly when engaging LTC residents who are often marginalized in decision-making. A key aspect of the RTA approach is viewing interviews as a reciprocal relationship between interviewer and interviewee, within which both parties shape data collection. The fact that members of the research team came from a diverse range of positionalities as LTC researchers, including one investigator who is a physician in LTC and several with family members in LTC, is a strength of this work as it allowed us to engage with a variety of experiences in data collection, as well as during data analysis and interpretation of the findings. The research team met weekly to debrief and to reflect on how the positionalities shaped the research process. Despite these limitations, this study offers valuable insights into the lived experiences and decision support needs of residents, care partners, and staff during care transitions, and contributes to the growing body of literature aimed at improving person-centered care in LTC settings.

### Future implications

Building on the insights from this study, there is a clear need for the development of a decision preparation and support intervention tailored to LTC residents, care partners, and staff. Such a tool could support informed decision-making by offering clear, accessible information about available care options, the risks and benefits of hospital transitions, and the comparative resources available in LTC versus hospital settings. Importantly, it could also facilitate meaningful conversations about care preferences, helping to ensure that transitions align with residents’ values and goals. Future research should strive to include more diverse populations by recruiting from ethnically and culturally specific LTC homes or LTC homes in culturally diverse communities to develop a more comprehensive understanding of care transitions among LTC residents. Examining the experiences of residents and care partners from diverse backgrounds will inform the design of more inclusive and culturally responsive transition interventions. The findings of this study will inform a series of co-design workshops in LTC homes aimed at developing a decision support tool.

## Conclusion

Transitions from LTC to hospital are complex processes that can significantly impact the well-being of residents and care partners. This study highlights critical gaps in communication, continuity of care, decision-making, and trust, all of which undermine person-centered transitions. To improve transition experiences, a shift toward integrated, collaborative models of care that prioritize resident autonomy, transparent communication, and relational continuity is essential. Addressing ageism and promoting shared decision-making practices are also vital to supporting more intentional and values-aligned transitions. Finally, fostering trust through inclusive, culturally safe care environments where residents and care partners feel empowered to voice their concerns must become a central goal of transition planning. Future research should explore interventions that enhance interdisciplinary communication and examine how overnight staffing and physician availability influence transition outcomes. Together, these efforts can help ensure that transitions between care settings support the dignity, safety, and preferences of those who rely on them. Ultimately, this research lays the foundation for codesigning future interventions with LTC residents, care partners, and staff to strengthen preparedness and promote resident-centered decision-making during transitions to hospital care.

## Supplementary Material

gnaf272_Supplementary_Data

## Data Availability

The data sets generated and analyzed during this study are not publicly available due to the nature of the research containing potentially identifying and sensitive information. Study materials including the study protocol have been previously published (Ménard et al., 2024). Reporting is in accordance with the Standards for Reporting Qualitative Research (SRQR) guidelines. This study was not preregistered.
