# Supplementary material for: “The first transition is from resident to patient”: understanding the decisional needs of long-term care residents preparing for hospital transitions
Source: Gerontologist. 2025 Nov 21;65(12):gnaf272. doi: 10.1093/geront/gnaf272 (PMC12714383; doi:10.1093/geront/gnaf272)
Supplement: gnaf272_Supplementary_Data [file gnaf272_supplementary_data.pdf]

Title: "The First Transition is from Resident to Patient": Understanding the Decisional Needs of Long-Term Care Residents Preparing for Hospital Transitions

Alixe Ménard, MSc (alimenard@ohri.ca)[1], Yamini Singh, MPH (yasingh@ohri.ca)[1], Lauren Konikoff, MA (lkonikoff@bruyere.org)[2], Michaela Adams, MSc (madams@perleyhealth.ca)[3], Daniel Kobewka, MD (dkobewka@toh.ca)[1,2], Krystal Kehoe MacLeod, PhD (kmacleod@bruyere.org)[2,4].

1. Department of Medicine, Ottawa Hospital Research Institute, Ontario, Canada
2. Bruyère Health Research Institute, Ontario, Canada
3. Perley Health Centre of Excellence in Frailty-Informed Care™, Ontario, Canada
4. Department of Family Medicine, University of Ottawa, Ontario, Canada

Corresponding Author:

Alixe Ménard
1053 Carling Ave
Ottawa, ON K1Y 4E9
T: 613-798-5555, ext. 19341
E: alimenard@ohri.ca
X: @alixevmenard

Supplementary Material. Interview Guide for Residents.

Hi, my name is [NAME]. I am part of the research team at the Bruyère Health Research Institute working on a project called, “Supporting Long-term Care Residents Through Transitions to Acute Care Hospital”. Thank you again for agreeing to participate in this interview and for meeting with me today. This interview format is called semi-structured which means I'll guide it with questions but really, you are in charge. Since we just reviewed the informed consent form, you know that we are here to learn about your experience transitioning from LTC to hospital. Are you ready to begin?

1. Can you tell me a bit about your time living here in a long-term care (LTC) home?
    a. How long have you been living here?
    b. Where were you living before?
    c. What is it like living here?
    d. Do you have family members or people that are important to you that come visit with you? Who? How often?
2. During your time living here in LTC, have you visited the hospital?
    a. Why?
    b. How many times?
    c. Did a friend or family member come with you?
3. Let’s rewind to when you first came to live here in LTC. I want to talk about a specific time that you went from LTC to hospital and had to decide if you should go or not. Can you recall discussing with the care providers about going to the hospital?
    a. Can you tell me about what happened?
    b. Before even needing to visit the hospital, did you ever talk about what might happen that might require you to consider visiting the hospital?
    c. Did you have a plan in place for deciding whether to go to the hospital?
    d. How was this plan developed? Did you use a specific tool to develop this plan?
    e. Who was involved in making this plan? Friends? Family? Doctor?
    f. Do you feel that you were consulted/involved in this decision-making process about whether to go to hospital?
    g. Did you and your loved ones agree with this plan for deciding whether to go to the hospital? Explain.
    h. Do you understand the plan and what information will be used to decide if you should go to the hospital or not?

i. Do you feel as though you received enough information about options, benefits, and risks?

i. Did you feel as though as you received too much information at once?

4. Okay, let's move on to your most recent hospital visit. Did you follow your plan when deciding to go? Explain.
    a. If not (or if you don't have a plan) how was the decision made for you to visit the hospital? Did you use a specific tool to make this decision?
    b. Were you involved in this decision-making process?
    c. Was your family involved in the decision-making process?
    d. Can you explain the main reasons you went to the hospital?
    e. Looking back, was it a good idea to go to the hospital? Explain.
    f. What benefits did you get from visiting the hospital?
    g. What challenges did you experience because of the decision to visit the hospital?
    h. Were these possible benefits and challenges explained to you before you decided to go to the hospital? Who explained them?
    i. Do you feel you understood the benefits and challenges of going to hospital before or at the time of your transfer?
    j. If you had a chance to make the decision again, would you still choose to visit the hospital?
    k. Do you feel that there was an alternative to a hospital visit? Explain.
5. How do you think residents and family members could be better supported before and during decisions about transition from long-term care to hospital?
6. How do you think staff members could be better supported before and during decisions about transition from long-term care to hospital or end-of life care?
7. Is there anything else you wish to tell me about your experience making the decision to visit the hospital while living in a LTC home?
8. Is there anything else I should know?

Thank you so much for your time and for sharing your valuable insights with me. As mentioned in the consent form, I will follow up with you today about the $25 gift card we are sending you as a small token of appreciation for your participation in our research. Have a wonderful day!
